# Accelerated aortic 4D flow cardiovascular magnetic resonance using compressed sensing: applicability, validation and clinical integration

**DOI:** 10.1186/s12968-019-0573-0

**Published:** 2019-10-21

**Authors:** Elisabeth Neuhaus, Kilian Weiss, Rene Bastkowski, Jonas Koopmann, David Maintz, Daniel Giese

**Affiliations:** 1 Institute for Diagnostic and Interventional Radiology, University of Cologne, Faculty of Medicine and University Hospital of Cologne, Kerpener Str. 62, 50937 Cologne, Germany; 2 Philips GmbH, Hamburg, Germany

**Keywords:** 4D flow CMR, Aorta, Compressed sensing, SENSE, Phase-contrast CMR

## Abstract

**Background:**

Three-dimensional time-resolved phase-contrast cardiovascular magnetic resonance (4D flow CMR) enables the quantification and visualisation of blood flow, but its clinical applicability remains hampered by its long scan time. The aim of this study was to evaluate the use of compressed sensing (CS) with on-line reconstruction to accelerate the acquisition and reconstruction of 4D flow CMR of the thoracic aorta.

**Methods:**

4D flow CMR of the thoracic aorta was acquired in 20 healthy subjects using CS with acceleration factors ranging from 4 to 10. As a reference, conventional parallel imaging (SENSE) with acceleration factor 2 was used. Flow curves, net flows, peak flows and peak velocities were extracted from six contours along the aorta. To measure internal data consistency, a quantitative particle trace analysis was performed. Additionally, scan-rescan, inter- and intraobserver reproducibility were assessed. Subsequently, 4D flow CMR with CS factor 6 was acquired in 3 patients with differing aortopathies. The flow patterns resulting from particle trace visualisation were qualitatively analysed.

**Results:**

All collected data were successfully acquired and reconstructed on-line. The average acquisition time including respiratory navigator efficiency with CS factor 6 was 5:02 ± 2:23 min while reconstruction took approximately 9 min. For CS factors of 8 or less, mean differences in net flow, peak flow and peak velocity as compared to SENSE were below 2.2 ± 7.8 ml/cycle, 4.6 ± 25.2 ml/s and − 7.9 ± 13.0 cm/s, respectively. For a CS factor of 10 differences reached 5.4 ± 8.0 ml/cycle, 14.4 ± 28.3 ml/s and − 4.0 ± 12.2 cm/s. Scan-rescan analysis yielded mean differences in net flow of − 0.7 ± 4.9 ml/cycle for SENSE and − 0.2 ± 8.5 ml/cycle for CS factor of 6.

**Conclusions:**

A six- to eightfold acceleration of 4D flow CMR using CS is feasible. Up to a CS acceleration rate of 6, no statistically significant differences in measured flow parameters could be observed with respect to the reference technique. Acquisitions in patients with aortopathies confirm the potential to integrate the proposed method in a clinical routine setting, whereby its main benefits are scan-time savings and direct on-line reconstruction.

**Electronic supplementary material:**

The online version of this article (10.1186/s12968-019-0573-0) contains supplementary material, which is available to authorized users.

## Background

The assessment of cardiovascular haemodynamics is important for the diagnosis and monitoring of many cardiovascular diseases including valvular heart diseases, congenital heart defects and aortopathies. In addition to echocardiography, phase-contrast (PC) cardiovascular magnetic resonance imaging (CMR) provides a tool to non-invasively quantify blood flow [1]. 4D flow CMR allows the assessment of the 3D, three-directional, time-resolved blood flow [2]. As compared to 2D PC-CMR, planning is simplified and a retrospective quantification of flow parameters in any desired imaging plane within the acquired volume makes 4D flow CMR less operator dependent [2]. However, as compared to 2D PC-CMR which is widely available and used in standardised CMR protocols [3, 4], 4D flow CMR currently remains technically challenging and is rarely used in clinical routine settings. This is mainly due to the inherently long scan time which is further increased by the need for respiratory motion compensation [5]. An aortic acquisition with parameters as described in the 4D flow CMR consensus statement [6] takes approximately 15 min by using standard parallel imaging techniques on a typical clinical CMR system. Therefore, a range of acceleration techniques that exploit spatio-temporal correlations have been applied to 4D flow CMR of the heart and/or surrounding vessels including *k-t* BLAST [7], *k-t* GRAPPA [8, 9] or *k-t* PCA [10, 11] allowing for acceleration factors in the order of 5 to 8. Acceleration techniques based on compressed sensing (CS) − often combined with non-Cartesian sampling, with and without exploitation of spatio-temporal correlations − have also been applied to 4D flow CMR achieving similar acceleration rates [12–15]. One major disadvantage of most proposed CS techniques is their relatively long off-line reconstruction time, reportedly being in the range of 45 to 60 min [12, 13, 16].

The aim of this study was to systematically evaluate the use of a CS technique, combining CS and parallel imaging, including on-line reconstruction to accelerate the acquisition and reconstruction of 4D flow CMR of the thoracic aorta. The impact of different CS acceleration factors on the measurement of direct haemodynamic parameters including net flow (NF), peak flow (PF) and peak velocity (PV) was assessed in healthy subjects. As a reference, conventional parallel imaging 4D flow CMR was used. A scan-rescan and inter- and intraobserver analysis were additionally performed on these parameters.

Finally, the proposed CS 4D flow CMR technique was applied to three patients with bicuspid aortic valves or Marfan syndrome to show feasibility in a clinical routine CMR examination of diseases in which aortic haemodynamic changes are relevant [17–20].

## Methods

### Study population

Between June 2018 and January 2019, 20 healthy subjects (27.1 years, range: 21–41; 11 women) without known cardiovascular diseases and 3 patients with aortopathies (further characterised in Table 1) were recruited. The study was approved by the local ethics committee and written informed consent was obtained from all participants.

**Table 1:** Patient characteristics

Patient #	Age [y]	Sex	Disease
1	49	f	Bicuspid aortic valve (Sievers’ type 1/LR) with ascending aortic dilatation
2	33	f	Bicuspid aortic valve (Sievers’ type 1/LR) with aortic insufficiency and ascending aortic dilatation
3	39	f	Marfan syndrome, dilatation of the aortic annulus and sinus

### CMR imaging

#### Healthy subjects

4D flow CMR in healthy subjects was acquired on a clinical 3 T CMR system (Ingenia; Philips Healthcare, Best, The Netherlands) with a 28-channel coil array. A sagittal volume covering the thoracic aorta was acquired. No contrast agent was used. Acquisition parameters were chosen in accordance with the consensus statement [6] and are listed in Table 2. A non-symmetric four-point flow encoding scheme was used, generating one flow-compensated and three flow-encoded acquisitions [21]. All acceleration factors were defined as scan time reductions with respect to a fully sampled dataset. The number of healthy subjects was divided into two sub-groups, group 1 consisting of 15 subjects and group 2 consisting of 5 subjects.

**Table 2:** Acquisition parameters

	Healthy Subjects		Patients
Repetition time [ms]	3.5		2.9
Echo time [ms]	2.2		1.7
Field of View [mm^3^]	280 x [220–290] x [45–60]		280 x [280–300] × 90
Acquisition voxel size [mm^3^]		2.5 × 2.5 × 2.5	
Recon voxel size [mm^3^]		1.25 × 1.25 × 1.25	
Flip angle [degrees]	4		10
Velocity encoding [cm/s]		150	
Acquired temporal resolution [ms]	49.3 ± 7.8		52.0 ± 1.9
Reconstructed temporal resolution [ms]	39.4 ± 6.2		39.0 ± 1.4
Acceleration mode/ factors *R*	SENSE: *R* = 2, (6)		CS: *R* = 6
	CS: *R* = 4, 6, 8, 10		

Acquisition parameters differ slightly between volunteer and patient study for two reasons: 1) patient measurements were performed after the administering of contrast agent which necessitate the increase of flip angle, 2) patients with dilatation of the aorta require a larger field of view to cover the aorta. Both modifications effect repetition and echo time

In group 1, five or six 4D flow CMR acquisitions were performed per subject: four CS (acceleration factors R = 4, 6, 8 and 10 - termed CS4, CS6, CS8 and CS10) and one parallel imaging (SENSE acceleration factor R = 2 - termed S2) sequences were acquired. In addition, 9 subjects further underwent an acquisition with a SENSE acceleration factor of R = 6 (S6). To decrease the probability of systematic effects due to long examination lengths such as heart rate variations and motion, acquisitions were performed in a randomised order for each subject (except for the S6 scan, which was always acquired last).

In group 2, the scan-rescan reproducibility of the reference technique (S2) and of CS6 and CS8 was assessed using identical acquisition parameters as in group 1. The scans were also performed in a randomised order for each subject and repeated in the same order after a short break including repositioning and replanning.

#### Patients

Three patients were acquired on a clinical 1.5 T CMR system (Ingenia, Philips Healthcare) with a 28-channel coil array. The patients underwent a clinically indicated standard-of-care CMR examination, complemented by a CS6 4D flow measurement. As all patients underwent a contrast-enhanced angiography, the flip angle of the subsequent 4D flow CMR acquisition was increased to account for reduced longitudinal relaxation times in the blood pool (Table 2).

Coil compression [22] to 4 virtual coils was used to accelerate reconstruction times. For the S6 scan, coil compression to 12 virtual coils was used.

All acquisitions were triggered retrospectively to the electrocardiogram (ECG) and a pencil-beam respiratory navigator was placed on the right diaphragm-liver interface. The acceptance window for respiratory gating was set to 6 mm.

The CS technique used in this work combines the compressed sensing and the parallel imaging (SENSE) approaches. The CS as well as the used SENSE technique were provided by the manufacturer as product sequences (Compressed SENSE/ SENSE, Philips Healthcare). In line with most CS techniques, a variable density incoherent undersampling pattern with a more densely sampled *k*-space centre was used. A regularised, iterative L1 norm minimisation was then performed assuring image sparsity in the wavelet domain and data consistency [23–25]. Each time frame was reconstructed separately and no temporal correlations were exploited in the reconstruction. Reconstruction was CPU-parallelised on the standard reconstruction workstation of the CMR systems (32 GB RAM, Intel Xeon E5–1620 CPU).

### Data analysis

Image reconstruction and concomitant field correction were performed on-line [26] while eddy current induced background phase correction [27], velocity un-aliasing and all post-processing steps were performed off-line using GTFlow (version 3.1, Gyrotools LLC, Zurich, Switzerland).

#### Healthy subjects

For each subject, six 2D planes were manually placed in the S2 scans. All planes intersected the aorta orthogonally to the main flow direction: one at the aortic root (Root), one at the ascending aorta (AAo), two at the aortic arch (Arch1, Arch2) and two at the descending aorta, the latter at the level of the lung-liver interface (DAo1, DAo2) (see Fig. 2). For each of these planes, contours were manually adjusted for each time point in order to compensate for aortic in-plane motion and deformation throughout the cardiac cycle. Subsequently, the contours were transferred to the CS accelerated scans. All contours were re-adjusted in the in-plane dimensions to compensate for possible bulk motion between acquisitions while their shape was kept unchanged. NF and PF were extracted from all contours whereas PV was solely extracted from the contour Root.

To assess inter- and intraobserver variability, NF and PF were additionally extracted from AAo and DAo1 in the S2 and CS6 scans from contours drawn by a second blinded observer and re-drawn by observer 1.

Finally, a quantitative particle trace analysis was performed to test for internal data consistency based on the conservation-of-mass principle. For this purpose, the percentage of particles emitted from contour AAo that reached contour DAo1 was measured. In average 398 particles were released during five time-points in early systole (time interval: 31.5 ms). Arriving particles were counted during one R-R interval.

#### Patients

In patients with aortopathies, a qualitative analysis of blood flow patterns was performed by particle trace visualisation and compared to previously described flow patterns in the respective diseases.

### Statistical analysis

In the following, all values are given as mean ± standard deviation (SD). Bland-Altman analyses of NF, PF and PV were performed to evaluate the variability between the S2 and the CS accelerated acquisitions. Similarly, Bland-Altman analyses were performed for inter- and intraobserver variability analysis. 95% confidence intervals (CI) were calculated for mean differences (MD) and for limits of agreements (as defined by MD ± 1.96 * SD). To allow a more sensitive measure of potential temporal deviations between flow curves, the time-accumulated flow error, averaged over all contours and subjects, was calculated [10]:

$$ {E}_R=\frac{1}{n_S}\sum \limits_{S=1}^{n_S}\left(\frac{1}{n_C}\sum \limits_{C=1}^{n_C}\left(\frac{\sum \limits_{t=0}^{t_{n_T}}\left|{Q_{\mathrm{S}2}}_{S,C}(t)-{Q_{\mathrm{CS}\ \mathrm{R}}}_{S,C}(t)\right|}{\sum \limits_{t=0}^{t_{n_T}}{Q_{\mathrm{S}2}}_{S,C}(t)}\right)\right) $$

where *n*_*S*_*, n*_*C*_ and *n*_*T*_ correspond to the number of subjects, contours and time frames, respectively. *Q*_S2*S*, *C*_(*t*) and *Q*_CS R*S*, *C*_(*t*) denote the flow rates through the contour C of subject S at time-point t in the S2 and CS factor R measurements (R = 4, 6, 8, 10), respectively.

## Results

All healthy subjects and patient data were successfully acquired and reconstructed on-line. None of the healthy subject scans needed to be aborted nor repeated. Reconstruction times were approximately 9 min for a CS6 sequence.

### Healthy subjects

In 9 subjects, the examination could be extended by the additional S6 acquisition. The acquisition times including navigator efficiency, averaged over all subjects of group 1, were 15:09 (S2), 6:48 (CS4), 5:02 (CS6), 3:33 (CS8) and 2:52 min (CS10). With respect to the S2 acquisition, this corresponds to a 55, 67, 77 and 81% scan time reduction for CS4, CS6, CS8 and CS10, respectively (for absolute values, see Table 3). The mean respiratory gating efficiency was 51 ± 10%.

**Table 3:** Acquisition time, net flow, peak flow and peak velocity averaged over all healthy subjects of group 1 for different SENSE and CS acceleration factors

	S2	CS4	CS6	CS8	CS10	S6
Acquisition time excluding (including) gating efficiency [min]						
	7:32 ± 1:29	3:20 ± 0:38	2:18 ± 0:26	1:41 ± 0:17	1:22 ± 0:16	2:00 ± 0:11
	(15:09 ± 5:19)	(6:48 ± 2:12)	(5:02 ± 2:23)	(3:33 ± 1:11)	(2:52 ± 0:53)	(3:48 ± 0:44)
Net flow [ml/cycle]						
Root	88 ± 14	91 ± 16	89 ± 13	87 ± 14	84 ± 17	86 ± 12
AAo	87 ± 15	86 ± 13	85 ± 16	84 ± 14	81 ± 13	78 ± 14
Arch1	89 ± 16	81 ± 14	84 ± 16	82 ± 13	79 ± 15	76 ± 21
Arch2	61 ± 11	61 ± 11	60 ± 14	60 ± 10	59 ± 11	70 ± 14
DAo1	58 ± 11	59 ± 11	59 ± 13	59 ± 10	57 ± 10	58 ± 10
DAo2	55 ± 11	53 ± 10	54 ± 12	54 ± 11	46 ± 11	45 ± 11
Peak flow [ml/s]						
Root	407 ± 56	415 ± 60	411 ± 56	409 ± 58	401 ± 65	398 ± 48
AAo	398 ± 58	392 ± 53	389 ± 59	391 ± 63	376 ± 53	361 ± 40
Arch1	376 ± 62	358 ± 56	365 ± 57	362 ± 55	438 ± 51	340 ± 48
Arch2	264 ± 37	265 ± 36	259 ± 47	258 ± 40	261 ± 42	284 ± 34
DAo1	257 ± 34	257 ± 33	260 ± 37	258 ± 36	254 ± 39	245 ± 28
DAo2	226 ± 35	225 ± 32	226 ± 43	223 ± 35	201 ± 39	195 ± 36
Peak velocity [cm/s]						
Root	130 ± 8	135 ± 9	137 ± 12	138 ± 15	134 ± 12	159 ± 19

*AAo* ascending aorta, *CS* compressed sensing, *DAo* descending aorta

**Fig. 1:**
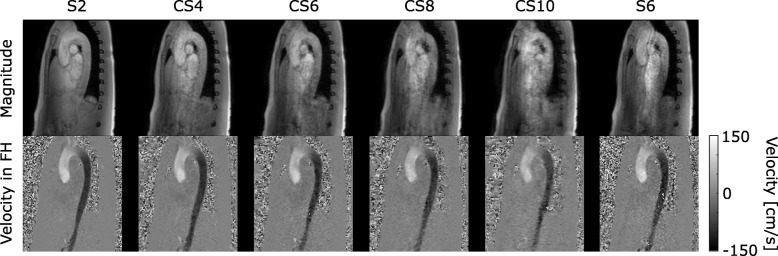
Magnitude and phase-contrast images. Sagittal slices through the aorta of an exemplary dataset in peak systole. The different acceleration techniques and factors are shown in columns and labelled correspondingly. Magnitude (top row) and phase-contrast (bottom row) images with velocity encoding in foot-head direction (FH) are shown. Increased image artefacts can be observed for higher acceleration factors

**Fig. 2:**
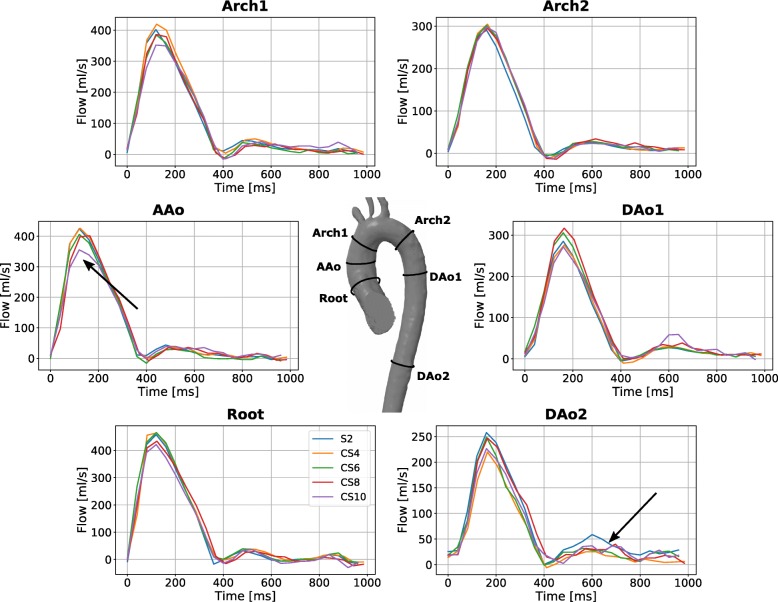
Net flow curves. Exemplary net flow curves extracted from datasets acquired with different acceleration factors. Each subfigure represents a different contour within the aorta. The arrows depict stronger deviations between acceleration factors. Main differences between the flow curves can be observed during peak systole. The three-dimensional figure of the aorta in the center shows the five contours where haemodynamic parameters were evaluated

Exemplary magnitude and phase-contrast images as well as net flow curves show decreasing quality with increasing acceleration factor (Figs. 1 and 2). More caudal contours show stronger deviations (Fig. 2).

**Fig. 3:**
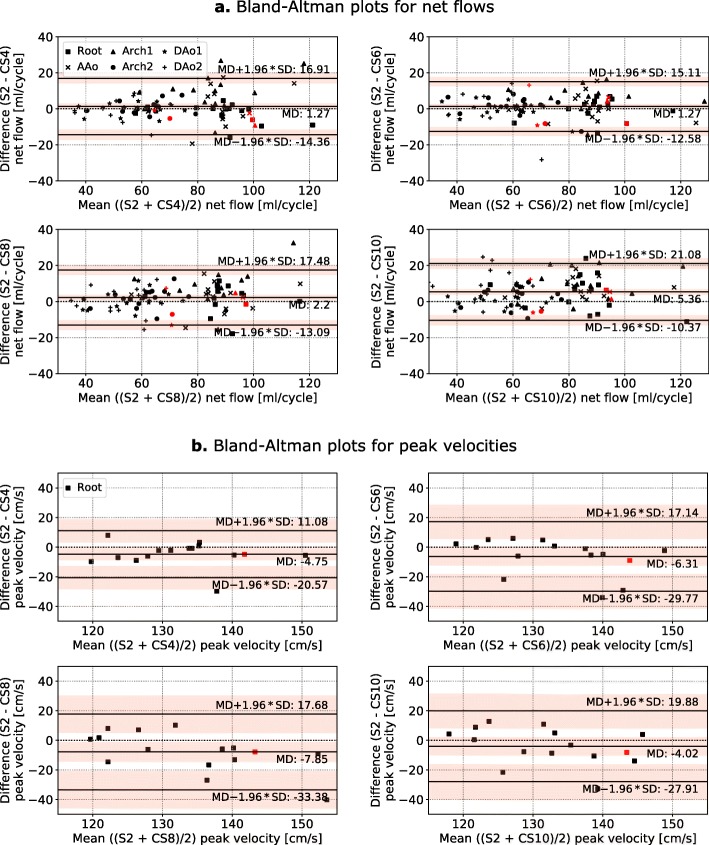
Bland-Altman analysis. Bland-Altman plots comparing (**a**) net flows in six contours and (**b**) peak velocities in the aortic root of S2 and compressed sensing (CS) accelerated scans. Data from different contours are indicated by different markers. The mean differences, standard deviations and their confidence intervals (red shaded areas) were calculated from the data points of all contours. The red data points denote the data of the volunteer whose net flow curves are shown in Fig. 2. The mean differences in net flow (**a**) between S2 and CS increase with increasing CS acceleration factor, while the standard deviations of the mean differences remain almost constant. The peak velocities (**b**) tend towards negative biases for all CS acceleration factors, while only exceeding the confidence interval of the mean difference for CS8. The standard deviation of the mean difference increases with increasing acceleration factor

These findings are confirmed by quantitative comparisons of NF, PF and PV as summarised in Table 3. Absolute discrepancies between S2 and CS8 averaged over the contours are 2.3 ml/cycle for NF 5.5 ml/s for PF and 8 cm/s for PV. Increasing deviations of NF and PF can be observed for CS10 (5.3 ml/cycle and 20.2 ml/s). A detailed analysis of MD, limits of agreement and CI is shown in the Bland-Altman plots in Fig. 3 and Additional file 1. All values are summarised in Table 4, revealing no significant differences in measured flow parameters up to a CS acceleration rate of 6 compared to S2. From an acceleration rate of 8, the NF shows a statistically significant underestimation of 2.2 ± 7.8 ml/cycle for CS8 and of 5.4 ± 8.0 ml/cycle for CS10. A significant underestimation of the PF was found for CS10 (14.4 ± 28.3 ml/s). In contrast to NF and PF, Bland-Altman analysis of PV tends towards negative biases for all acceleration factors (on average − 4%), which only for CS8 exceeds the CI of the MD. The SD of the MD show a continuous increase from CS4 to CS10 for PF (from 20.4 ml/s to 28.3 ml/s) and PV (from 8.1 cm/s to 12.2 cm/s).

**Table 4:** Summary of results of Bland-Altman analyses

Comparison	Net Flow [ml/cycle]		Peak Flow [ml/s]		Peak Velocity [cm/s]	
	MD ± SD	CI of the MD	MD ± SD	CI of the MD	MD ± SD	CI of the MD
Intertechnique						
S2 vs. CS4	1.3 ± 8.0	(− 0.4; 2.9)	2.7 ± 20.4	(− 1.5; 6.9)	− 4.8 ± 8.1	(− 8.8; 0.7)
S2 vs. CS6	1.3 ± 7.1	(− 0.2; 2.7)	3.1 ± 22.3	(− 1.5; 7.7)	− 6.3 ± 12.0	(− 12.4; 0.3)
S2 vs. CS8	2.2 ± 7.8	(0.6; 3.8)	4.6 ± 25.2	(− 0.6; 9.9)	−7.9 ± 13.0	(− 14.4; − 1.3)
S2 vs. CS10	5.4 ± 8.0	(3.7; 7.0)	14.4 ± 28.3	(8.6; 20.3)	− 4.0 ± 12.2	(− 10.2; 2.2)
Inter-Observer^a^						
S2_1 vs. S2_2	−1.3 ± 2.6	(− 2.2; − 0.3)	−1.1 ± 6.7	(− 3.5; 1.3)		
CS6_1 vs. CS6_2	−0.4 ± 2.8	(− 1.4; 0.7)	2.1 ± 9.5	(−1.3; 5.5)		
Intra-Observer^a^						
S2_1 vs. S2_2	−0.4 ± 1.9	(−1.1; 0.2)	−0.7 ± 8.1	(− 3.6; 2.2)		
CS6_1 vs. CS6_2	−0.2 ± 2.3	(− 1.1; 0.6)	− 2.1 ± 10.7	(− 6.0; 1.7)		
Scan-Rescan^b^						
S2_1 vs. S2_2	− 0.7 ± 4.9	(− 2.4; 1.1)	1.9 ± 26.3	(− 7.5; 11.3)	5.1 ± 4.9	(0.9; 9.4)
CS6_1 vs. CS6_2	− 0.2 ± 8.5	(−3.2; 2.9)	4.8 ± 32.1	(− 6.7; 16.2)	2.1 ± 11.6	(− 8.0; 12.3)
CS8_1 vs. CS8_2	− 1.5 ± 8.9	(−4.7; 1.7)	6.9 ± 42.5	(− 8.3; 22.1)	14.8 ± 38.2	(− 18.7; 48.3)

Data are given as mean difference (MD) ± standard deviation (SD) and 95% confidence interval (CI) of the mean differences. The main data sample considers 6 contours from each of in total 15 subjects. ^a^ Results for inter- and intraobserver analysis were only extracted from AAo and DAo1. ^b^ Scan-rescan measurements were performed in 5 volunteers

**Fig. 4:**
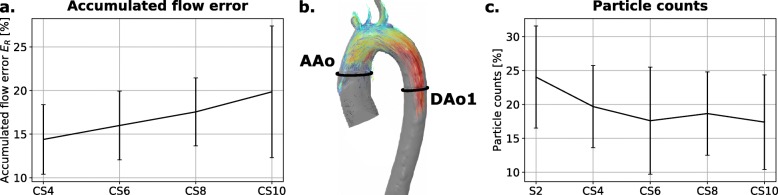
Advanced flow parameters. **a** Accumulated flow error E_R_ as a function of CS acceleration factor. **b** Exemplary particle trace visualisation including the contours from which particles were ejected (ascending aorta, AAo) and arriving particles were counted (descending aorta, DAo1). **c** Percentage of particles ejected from the contour AAo reaching the contour DAo1 as a function of the acceleration factor

In addition to the clinically relevant parameters NF, PF and PV, the accumulated flow error *E*_*R*_ constantly increases with increasing acceleration factor (Fig. 4a). Particle trace analysis demonstrates an increased loss of particles emitted from the ascending aorta reaching the descending aorta for higher CS factors while the main difference is seen between the S2 and the CS4 scans (Fig. 4b).

The Bland-Altman analysis of scan-rescan reproducibility demonstrates deviations for NF, PF and PV below 2.5% for all acceleration factors, while the deviations are lowest for S2 and increase with increasing acceleration factors CS6 and CS8 (Table 4, Additional file 2).

Inter- and intraobserver variabilities are similar for S2 and CS6 scans. Overall MD and SD of extracted flow parameters are under 1.6 and 4.2%, respectively (Table 4, Additional file 3).

### Patients

**Fig. 5:**
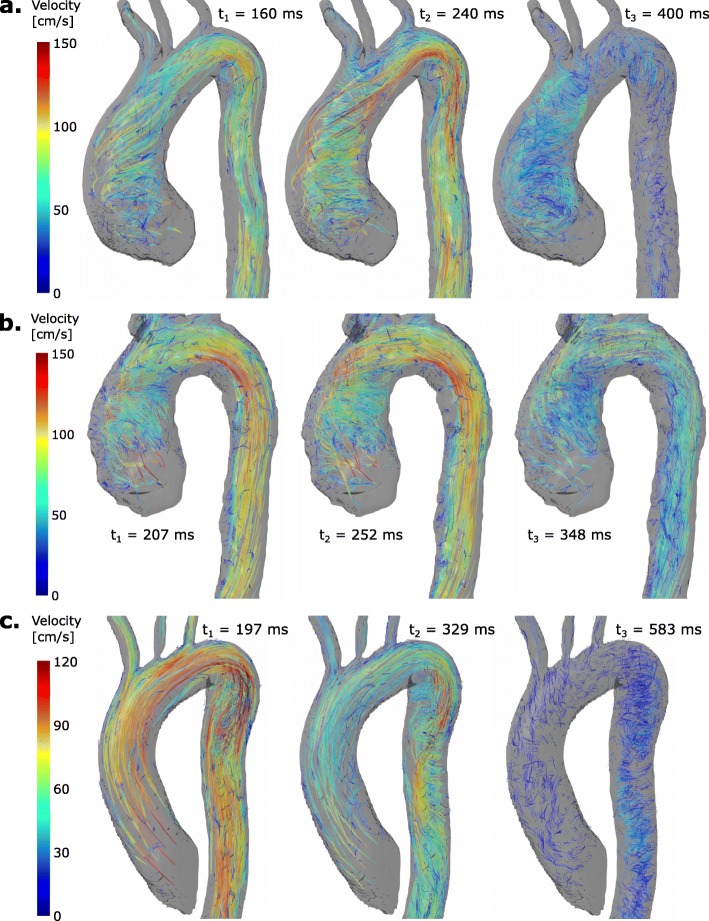
Pathline screenshots. Particle traces ejected from the aorta show aberrant flow patterns in patients #1 and #2 with bicuspid aortic valves (**a** and **b**) and patient #3 with Marfan syndrome (**c**). Patients #1 and #2 show pronounced right-handed helical flow patterns in the AAo; a smaller, slower helix is superimposed by a larger, faster and less-twisted helix. In patient #3 a helical flow along the DAo can be observed. For the corresponding movies please refer to the Additional files 4, 5 and 6


The additional time needed for the 4D flow CMR acquisitions within the clinical examination protocol was well tolerated by all patients. The blood flow patterns of all patients are illustrated in Fig. 5 for selected time points in the cardiac cycle. Time-resolved movies are available as Additional files 4, 5 and 6. Patients #1 and #2, both with bicuspid aortic valves and ascending aortic dilatations, show pronounced right-handed helical flow patterns in the AAo; a smaller, slower helix is superimposed by a bigger, faster and less-twisted helix. In case of patient #1, helical flow in the brachiocephalic trunk and a small global vortex at the inner curvature of the arch can also be observed. In patient #3 with Marfan syndrome a vortical flow pattern can be observed at the inner curvature of the transition from the arch to the proximal DAo as well as helical flow along the DAo.

**Additional file 4:** Pathline movie – Patient #1 (Bicuspid aortic valve). View of the aortic flow in patient #1 with bicuspid aortic valve. (MP4 16887 kb)



**Additional file 5:** Pathline movie – Patient #2 (Bicuspid aortic valve). View of the aortic flow in patient #2 with bicuspid aortic valve. (MP4 14391 kb)



**Additional file 6:** Pathline movie – Patient #3 (Marfan syndrome). View of the aortic flow in patient #3 with Marfan syndrome. (MP4 13540 kb)


## Discussion

This study evaluated the use of a combined CS and parallel imaging technique for the acceleration of 4D flow CMR in the thoracic aorta. A thorough quantitative analysis performed in healthy subjects demonstrates its feasibility for the assessment of direct haemodynamic parameters while exemplary patient acquisitions demonstrate its potential application in clinical CMR protocols. The used CS and SENSE techniques are based on product implementations from Philips Healthcare and are therefore readily available for the Philips CMR community.

Reconstruction times of approximately 9 min can remain limiting in some cases, but a concurrent reconstruction and further acquisition of subsequent CMR scans is possible. An off-line reconstruction on dedicated hardware is also possible and could increase reconstruction speed, but remains debatable in a clinical environment due to raw data transfer speed, laborious PACS-integration and potential hardware approval issues. Finally, an acceleration of on-line reconstruction times could also be achieved by making use of higher computational power devices, e.g. graphical processor units.

Although quality of the magnitude images visually suffers from increased artefacts for higher CS factors, all flow curves extracted from the CS 4D flow data compare well with the S2 reference dataset. Deviations of the resulting flow curves are in line with deviations of *k-t* accelerated 4D flow CMR measurements compared to standard accelerated 4D flow or 2D PC-CMR acquisitions [7, 8, 10].

Quantitative values such as NF, PF and PV show overall small deviations when compared to S2 scans, while a trend showing increased MD for higher CS factors can be observed. Up to a CS acceleration rate of 6, Bland-Altman analyses show no significant differences in measured flow parameters. In contrast to NF and PF, Bland-Altman analysis of PV tends towards negative biases for all acceleration factors (on average − 4%), which only for CS8 exceeds the CI of the MD. The used CS sequence slightly overestimates the PV. This effect can be ascribed to higher noise values and therewith higher maximal velocities appearing in the contours.

In contrast to other studies showing increased temporal smoothing and therewith decreased PF and PV for higher acceleration rates [8, 28–30], this effect is not present in this approach, as no temporal correlations are used in the CS reconstruction. The potential influence of physiological variations was minimised by the use of a randomised acquisition order. Remaining eddy current related phase offsets [31] or gradient non-linearities [32] might be additional causes of the remaining systematic deviations in NF, PF and PV. However, as all parameters such as field of view, the velocity encoding gradient, repetition and echo times were kept constant between all acquisitions, an influence of such effects can be considered minimal.

The limits of agreement for all analysed flow parameters are in-line with previous studies [8, 10].

The results for the accumulated flow error *E*_*R*_, which is increasing with a slightly larger slope between CS8 and CS10, are consistent with the obtained results from the Bland-Altman analyses. This error metric reaches nearly 20% and implies that the acquisition of net flow at a single time frame is very sensitive to scan acceleration. Moreover, the large standard deviations in these error metrics could be attributed to additional uncertainties including physiological differences between healthy subjects, contour size, cardiac cycle length, distance between contours and flow velocities.

The increased particle loss for higher CS factors with the main difference between the S2 and the CS4 scans, is due to the higher undersampling. The spatial distribution of undersampling artefacts induced by the CS reconstruction might be an additional effect: depending on regularisation, noise-like artefacts may be increased leading to increased particle losses. A divergence-free reconstruction constraint is expected to minimise these artefacts while assuring physical properties of flowing blood [33–35].

An assessment of scan-rescan reproducibility and of inter- and intraobserver variabilities were performed to better classify the findings obtained from S2 versus CS scans. The scan-rescan reproducibility was highest for S2 and decreased with increasing acceleration factors CS6 and CS8 while the effect on the different, measured haemodynamic parameters is comparable and in line with findings from previous studies [36–38]. Inter- and intraobserver variabilities are similar for S2 and CS6 scans. Overall MD and SD of extracted flow parameters are in line with previously described results [36]. Furthermore, they remain small when compared to the scan-rescan differences.

Based on the healthy subject data, a relatively conservative CS factor of 6 was chosen for the patient acquisitions. The net scan times of aortic 4D flow CMR acquisition were in the order of 2 min resulting in total scan times of approximately 5 min, depending on heart rate and breathing navigator efficiency. The observed flow patterns in all three patients are in good agreement with previously described results: For instance, abnormal helical flow in the AAo in patients with bicuspid aortic valves, depending on the valvular phenotype has been described [19, 20]. Similarly, a significantly increased helical flow as well as a more frequently occurrence of vortical flow in the DAo in Marfan syndrome patients has been reported [17, 18]. Data quality achieved with CS therefore allows the measurement of these distinct haemodynamic features.

Although this study investigates a limited group size of 23 subjects (20 healthy subjects, 3 patients), the results demonstrate that CS allows a 4 to 8-fold acceleration of 4D flow CMR, corresponding to another 2 to 4-fold acceleration compared to typically used parallel imaging factors (S2) and is ready for clinical use for the assessment of primary haemodynamic parameters. The benefit in scan-time can in the future be traded, dependent on the clinical question, for shorter examination times, higher spatial and/or temporal resolution or acquiring several velocity-encoded volumes (Multi-Venc 4D flow CMR)[42]. Regarding advanced higher-order metrics such as wall shear stress, pulse wave velocities or pressure gradients, we hypothesize that a CS accelerated scan can be treated as a standard 4D flow scan with noise increasing with acceleration factors. As higher-order metrics are known to rely on high spatial, temporal and/or velocity resolutions, an improved calculation of these parameters might be achieved by using CS to allow for higher resolutions at constant scan time. However, this effect was not evaluated in the current study and needs to be investigated in future studies.

One limitation of the presented work is that the healthy subject study was carried out at a 3 T CMR system whereas the patients were scanned during a clinical routine CMR examination at a 1.5 T system. Apart from the field strength, both systems are from the same generation, used the same acquisition and reconstruction software and have identical coil configurations. While 3 T provides a higher signal-to-noise-ratio (SNR), the patient scans were performed after administration of contrast agent with an increased flip angle, increasing the SNR in the patient measurement. As shown by Hess et al. [39] similar results of data acquired at 1.5 T with contrast agent and at 3 T without contrast agent can be expected.

The current evaluation was focussed on aortic 4D flow CMR. Further studies are needed before transferring CS accelerated 4D flow CMR to other vascular beds and areas of interest with other velocity ranges and/or spatio-temporal dimensions.

Finally, dedicated CS approaches for velocity and phase images including exploiting spatio-temporal data redundancy, velocity-encoding-dimension sparsity constraints or physical priors are warranted to allow improved image quality and higher acceleration techniques [11, 33–35, 40, 41].

## Conclusions

In summary, this study demonstrates that a six- to eightfold acceleration of 4D flow CMR using CS is feasible. Acquisitions in patients with aortopathies confirm the potential to integrate the proposed method in a clinical routine setting, whereby its main benefits are scan-time savings and direct on-line reconstruction.

## Additional files

Additional file 1: Bland-Altman analysis of peak flows. Bland-Altman plots comparing peak flows of S2 and CS accelerated scans in six contours (indicated by different markers). The mean differences, standard deviations and their confidence intervals (red shaded areas) were calculated from the data points of all contours. The red data points denote the data of the volunteer whose net flow curves are shown in Fig. 2. (PDF 243 kb)

Additional file 2: Bland-Altman analysis of scan-rescan agreement. Bland-Altman plots comparing (a) net flows, (b) peak flows and (c) peak velocities of in each case two repeated measurements of S2, CS6 and CS8 accelerated scans. The mean differences, standard deviations and their confidence intervals (red shaded areas) were calculated from the data points of all contours. (PDF 414 kb)

Additional file 3: Bland-Altman analysis of inter- and intraobserver agreement. Bland-Altman plots comparing net flows and peak flows resulting from inter- and intraobserver analysis. The mean differences, standard deviations and their confidence intervals (red shaded areas) were calculated from the data points of both contours. (PDF 370 kb)

## Data Availability

The imaging protocols as well as the datasets used and/or analysed during the current study are available from the corresponding author on reasonable request.

## Abbreviations

4D flow: Three-dimensional time-resolved phase-contrast magnetic resonance
AAo: Ascending aorta
Arch: Aortic arch
CI: Confidence interval
CMR: Cardiovascular magnetic resonance
CS: Compressed sensing
DAo: Descending aorta
ECG: Electrocardiogram
FH: Foot-head
MD: Mean difference
NF: Net Flow
PC: Phase-contrast
PF: Peak flow
PV: Peak velocity
Root: Aortic root
SD: Standard deviation
SENSE: Sensitivity encoding
SNR: Signal-to-noise-ratio
