# Supplementary figures and images for: Accelerated aortic 4D flow cardiovascular magnetic resonance using compressed sensing: applicability, validation and clinical integration
Source: J Cardiovasc Magn Reson. 2019 Oct 21;21:65. doi: 10.1186/s12968-019-0573-0 (PMC6802342; doi:10.1186/s12968-019-0573-0)

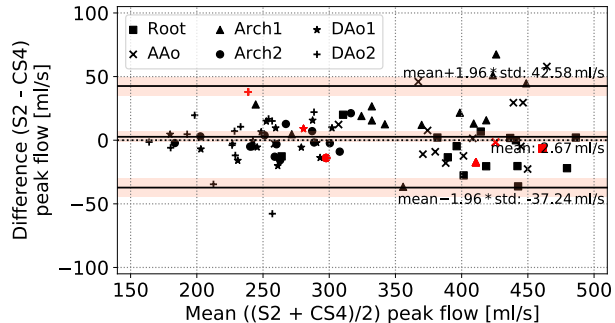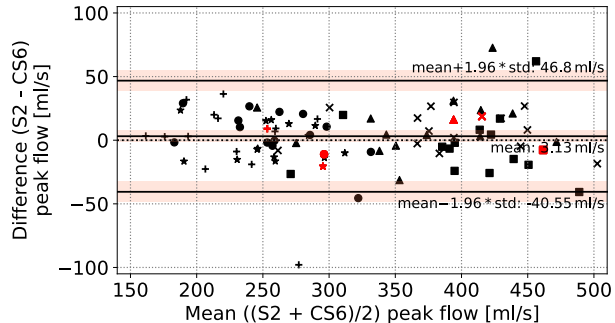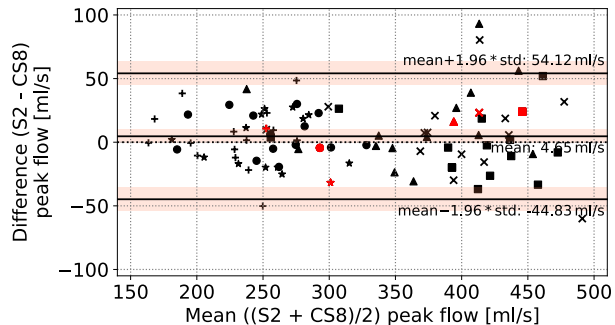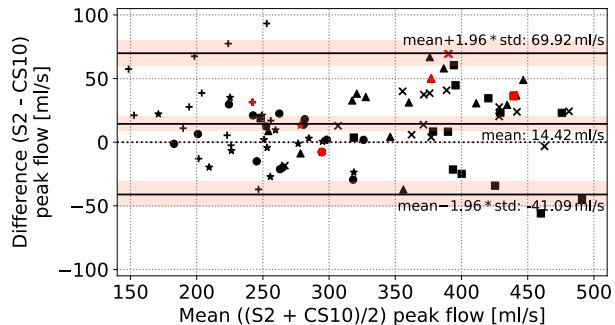

Supplement: Supplementary file 4 — Bland-Altman analysis of peak flows. Bland-Altman plots comparing peak flows of S2 and CS accelerated scans in six contours (indicated by different markers). The mean differences, standard deviations and their confidence intervals (red shaded areas) were calculated from the data points of all contours. The red data points denote the data of the volunteer whose net flow curves are shown in Fig. 2. (PDF 243 kb) [file 12968_2019_573_MOESM1_ESM.pdf]
