# Supplementary material for: Accelerated aortic 4D flow cardiovascular magnetic resonance using compressed sensing: applicability, validation and clinical integration
Source: J Cardiovasc Magn Reson. 2019 Oct 21;21:65. doi: 10.1186/s12968-019-0573-0 (PMC6802342; doi:10.1186/s12968-019-0573-0)

Scan-Rescan agreement

a. Bland-Altman plots for net flows

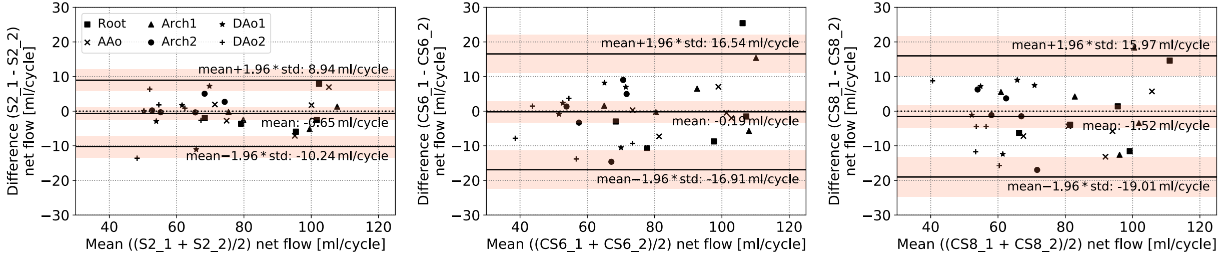

b. Bland-Altman plots for peak flows

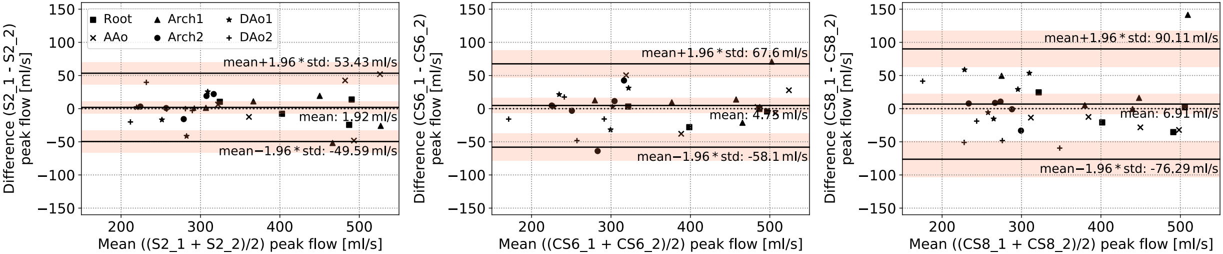

c. Bland-Altman plots for peak velocities

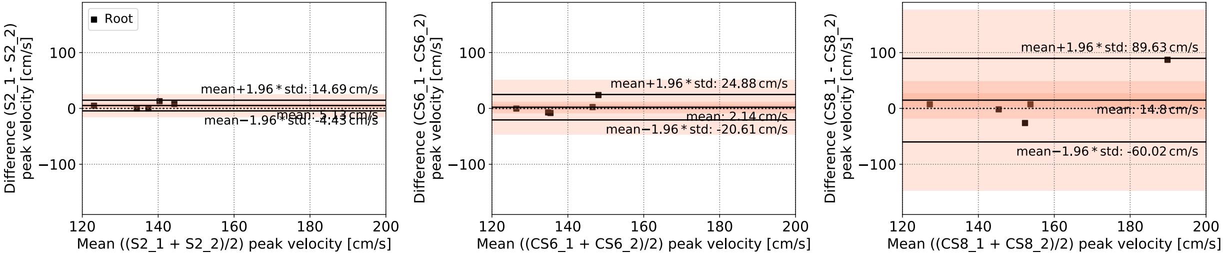

Supplement: Supplementary file 5 — Bland-Altman analysis of scan-rescan agreement. Bland-Altman plots comparing (a) net flows, (b) peak flows and (c) peak velocities of in each case two repeated measurements of S2, CS6 and CS8 accelerated scans. The mean differences, standard deviations and their confidence intervals (red shaded areas) were calculated from the data points of all contours. (PDF 414 kb) [file 12968_2019_573_MOESM2_ESM.pdf]
