# Supplementary material for: Accelerated aortic 4D flow cardiovascular magnetic resonance using compressed sensing: applicability, validation and clinical integration
Source: J Cardiovasc Magn Reson. 2019 Oct 21;21:65. doi: 10.1186/s12968-019-0573-0 (PMC6802342; doi:10.1186/s12968-019-0573-0)

# **1.** Interobserver Variability

## **a.** Bland-Altman plots for net flows

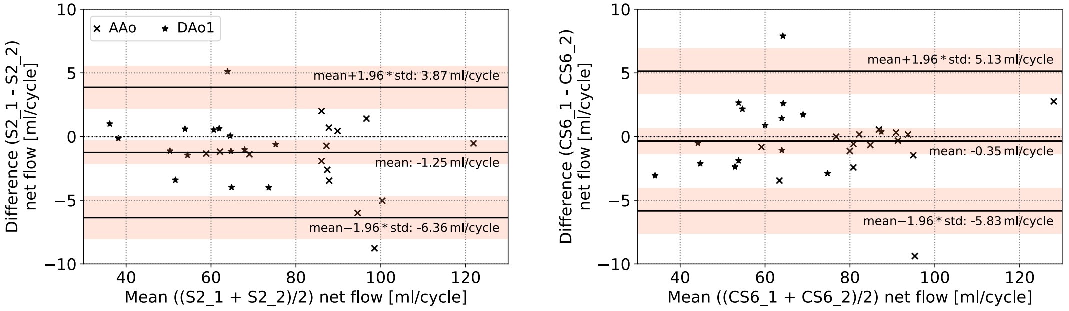


## **b.** Bland-Altman plots for peak flows

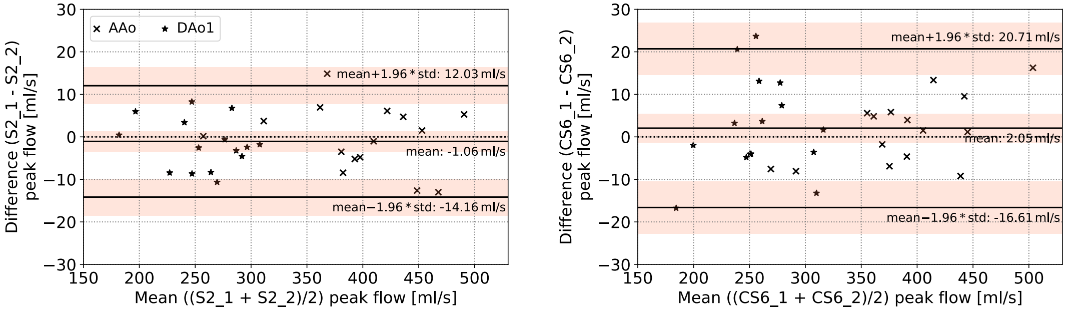


# **2.** Intraobserver Variability

## **a.** Bland-Altman plots for net flows

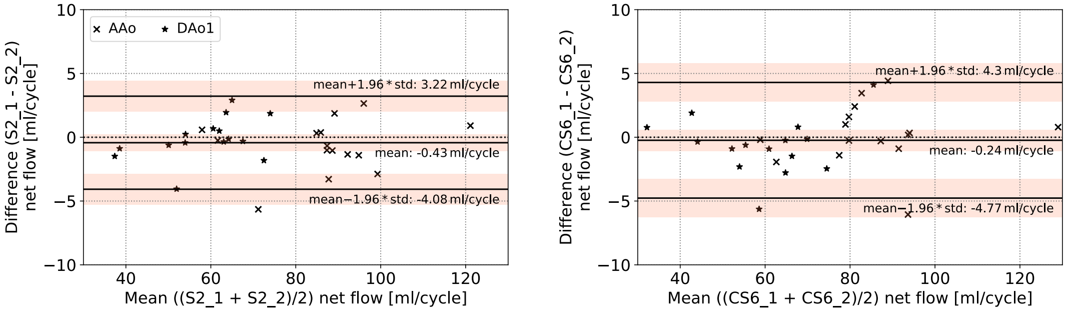


## **b.** Bland-Altman plots for peak flows

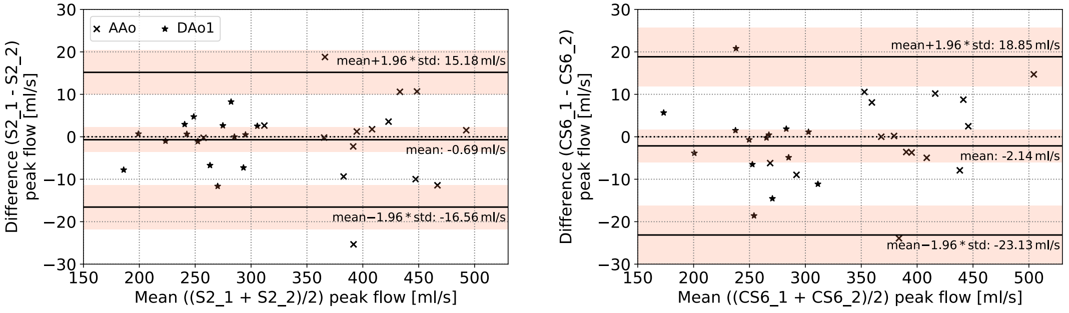

Supplement: Supplementary file 6 — Bland-Altman analysis of inter- and intraobserver agreement. Bland-Altman plots comparing net flows and peak flows resulting from inter- and intraobserver analysis. The mean differences, standard deviations and their confidence intervals (red shaded areas) were calculated from the data points of both contours. (PDF 370 kb) [file 12968_2019_573_MOESM3_ESM.pdf]
